# Anaerobic Digestion of Solid Agricultural Biomass in Leach-Bed Reactors

**DOI:** 10.3390/bioengineering10040433

**Published:** 2023-03-29

**Authors:** Ville Pyykkönen, Erika Winquist, Ari-Matti Seppänen, Markku Vainio, Elina Virkkunen, Kari Koppelmäki, Saija Rasi

**Affiliations:** 1 Production Systems, Natural Resources Institute Finland (Luke), P.O. Box 2, FI-00791 Helsinki, Finland; 2 Ruralia Institute, University of Helsinki, Lönnrotinkatu 7, FI-50100 Mikkeli, Finland

**Keywords:** agricultural biomass, biogas, leach-bed reactor, nutrient recycling

## Abstract

This study focuses on the feasibility of the dry anaerobic digestion of solid agricultural biomass for efficient renewable-energy production and nutrient recycling. Methane production and the amount of nitrogen in the digestates were measured in pilot- and farm-scale leach-bed reactors. In the pilot scale, with a digestion time of 133 days, the methane production of a mixture of whole crop fava bean and horse manure corresponded to 94% and 116%, respectively, of the methane potentials of the solid substrates. The mono-digestion of fava beans resulted in relatively low methane production (production/potential ratios of 59% and 57%). In two full-scale experiments, the methane production of mixtures of clover-grass silage, chicken manure, and horse manure corresponded to 108% and 100% of their respective methane potentials with digestion times of 117 and 185 days. In co-digestion, the production/potential ratios were similar in the pilot and farm experiments. High nitrogen loss was observed in the farm scale when the digestate was stored in a stack covered with a tarpaulin during summertime. Thus, although the technology seems promising, attention needs to be paid to management practices to minimise nitrogen losses and greenhouse gas emissions.

## 1. Introduction

The green transition and need to reduce the use of fossil fuels have been clear targets since the Paris agreement in 2015 [1] and the launch of the European Green Deal in 2019 [2]. In autumn 2021, the EU announced its Fit for 55 climate package that aims to speed up the reduction in greenhouse gas emissions, reducing them by 55% by 2030 compared to the levels in 1990 [3]. Moreover, from the beginning of 2022, the rise in energy prices has accelerated, and the availability of energy has become uncertain. Due to these challenges, there is a need to increase domestic renewable-energy production, including biogas production [4].

In Finland, almost the entire agricultural energy consumption (11 TWh) could be covered with domestic biogas production. However, current annual biogas production is only around 1 TWh [5]. Most of the unused potential lies in agricultural side streams and underutilised biomass such as grass, straw, and crop residues (72%), and livestock manure (14%) [6]. Especially in stockless organic crop farming, green manure and annual legumes are a substantial resource, as up to 60% of the cultivated area is used for the cultivation of nitrogen fixing plants. The use of green manure grass as the main feedstock in biogas production was demonstrated by Koppelmäki et al. [7]. This approach has several benefits. The multifunctional use of biomass where nitrogen fixing crops are part of the crop rotation can positively impact farming systems, resulting in enhanced nutrient cycling, carbon sequestration, and crop growth without competition between food and energy production [7,8,9,10,11,12]. During anaerobic digestion, part of the organic nitrogen in green biomass is converted into ammonium nitrogen, which is more easily available when used as fertiliser [9].

The most common technology in the anaerobic digestion of agricultural biomass is wet digestion, and the main raw material is slurry-type livestock manure [13]. In wet digestion, the total solids (TS) content in the reactor is usually limited to a maximum of 15% [14], as the digester content needs to be efficiently stirred and pumped. To be able to more extensively use underutilised solid agricultural biomass with over 20% TS in biogas production, dry digestion could be a suitable technology, but it is not as widely adopted in agriculture as it is in the treatment of municipal biowaste [15]. The main advantages of dry digestion include its simple and cost-efficient operation, low water requirement, the reduced need for the pretreatment of the feed material, not needing the solid–liquid separation of the digestate, and minimal nutrient loss [16,17]. Three main reactor types exist: the garage (batch), leach-bed (batch with percolation) and horizontal or vertical plug-flow (continuous) types [14,15,18,19].

Some problems reported in dry digestion are related to poor start-up performance, incomplete mixing, and the accumulation of volatile fatty acids (VFAs) [15]. The performance of dry digestion is improved in leach-bed reactors (LBRs) with the circulation of percolated liquid enabling more controlled and homogeneous conditions. LBRs are one-stage column reactors operated in batch mode through which percolated liquid collected at the bottom of the reactor is continuously recirculated to the top [20]. With this reactor type, Demirer and Chen [20] achieved around 25% improvement in biogas production in the laboratory scale with solid dairy manure compared to wet digestion.

This study partly fills the gap in the literature regarding the anaerobic digestion of different agricultural biomass types in leach-bed reactors. Not much research has been conducted on leach-bed reactors, especially in the farm scale. The aim of this study was to investigate the suitability of this technology in converting underutilised agricultural biomass into renewable energy and recycled fertilisers. Clover-grass silage and whole crop fava bean biomass was used as the main substrate in pilot- and farm-scale leach-bed reactors. Moreover, the beneficial impact of manure as the cosubstrate was studied.

## 2. Materials and Methods

### 2.1. Materials

Solid substrates used in the pilot-scale experiments were whole crop fava bean (FB) and horse manure (HM). The fava beans were harvested in Viitasaari, Finland and baled (plastic film) without preservatives. Horse manure (HM pilot) with sawdust as the bedding material was collected from a local stable. Raw materials used in the farm-scale experiments were clover-grass silage (CGS), chicken manure (CM), and horse manure (HM). The clover-grass was grown as green manure during summer 2018, Hyvinkää, Finland, harvested, and ensiled in a stack without preservatives (the same grass silage was used in both farm-scale batches in this study). Laying hen manure was obtained from a nearby henhouse, and horse manure from several stables in the area.

Inocula for the biochemical methane potential (BMP) experiments were obtained from a farm-scale wet digestion biogas plant treating cattle slurry (Natural Resources Institute Finland (Luke), Kuopio, Finland) at a mesophilic temperature (37 °C). Inocula for the BMP experiments were sieved to remove the coarse material before further treatment. The percolated liquid of farm-scale biogas plants treating grass silage and clover (Palopuro & Laukaa, Finland) was used as the inoculum in the pilot-scale experiments.

### 2.2. BMP and RMP Assays

Biochemical methane potential (BMP) assays were performed on all raw materials: fava beans (FBs), clover-grass silage (CGS), horse manure (HM), chicken manure (CM), and the percolation liquid used in the leach-bed reactor. Residual methane potential (RMP) assays were conducted on the solid digestates after the pilot- and farm-scale experiments, and on the percolation liquid of the pilot-scale experiments. All assays for all samples were performed in mesophilic (37 °C) conditions using automated testing equipment (Bioprocess Control Ltd., Lund, Sweden). The assays were mechanically mixed (84 rpm) for 1 minute per hour. The tests were conducted in 500 mL bottles (three parallel bottles for each sample and control (inoculum)). The raw material/inoculum VS:VS ratio varied depending on the experiment (Table 1). Distilled water was added to the bottles after the sample and the inoculum to achieve the desired liquid mass of 400 g. All bottles were buffered with NaHCO_3_ (3 g/L) and flushed with N_2_ to obtain anaerobic conditions. The CO_2_ of the produced biogas was absorbed in a 3M NaOH solution, and the volume of methane was determined with water displacement.

### 2.3. Pilot-Scale Experiments

#### 2.3.1. Pilot-Scale Reactor

**Figure 1:**
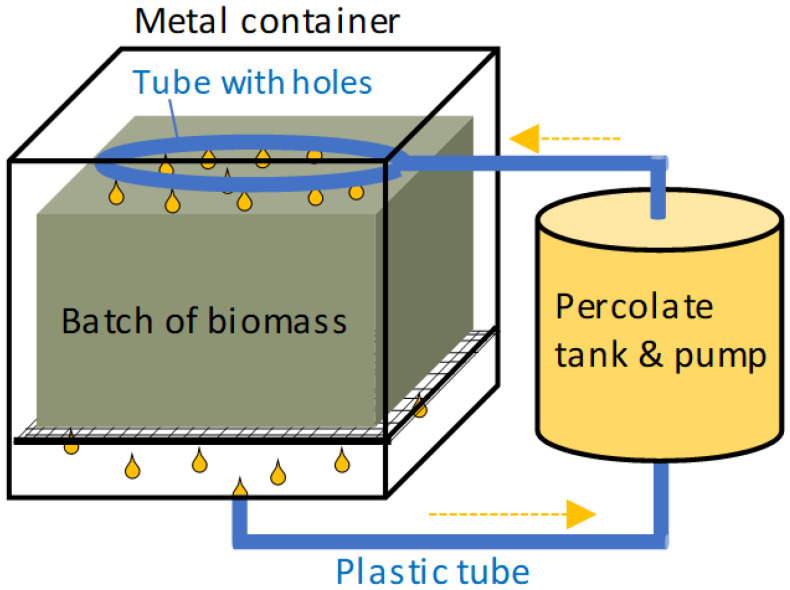
Schematic of the pilot-scale leach-bed reactor.

Pilot-scale experiments were performed in mesophilic (37 °C) conditions using two parallel 1 m^3^ leach-bed reactors (Metener Ltd., Leppävesi, Finland). Both reactors were equipped with their own percolation-liquid tanks (Figure 1). The percolation liquid (leachate) was circulated through the solid substrate. Depending on the stage of the process, the percolation liquid was circulated with varying flow rates from 0 to 60 L/d. The pH value of the percolation liquid was adjusted during the experiments using baking soda and 1 or 2 M NaOH until the pH was stable. The initial mass of the solid substrates and the percolation liquids is presented in Table 2. The temperature of the process was measured continuously. The produced volume of biogas and methane content, and pH were measured daily.

#### 2.3.2. Pilot-Scale Mass Balance Calculations

Mass balance for the pilot-scale experiments was calculated as follows. To calculate the amount of each mass component (ash, VS, TS, and nutrients) was added and removed, the contents of the solid feedstock and the percolate were analysed at the beginning and end of the experiment. The nutrient contents of the percolate that had been removed during the experiment were not analysed. Instead, the average of the initial and final percolate concentrations were used in the mass balance calculation. The mass of the produced biogas was calculated from volumetric gas flow data using densities of 0.716 kg/m^3^ for CH_4_ and 1.96 kg/m^3^ for CO_2_ (0 °C and 101.325 kPa). The mass fraction of the solid digestate in the total digestate (solid digestate + percolate) fresh matter (f_solid_) was calculated with Equation (1) (modified from [21]). The method assumes that the mass of ash does not change during the degradation of organic material:

f_solid_ = (C_ash total −_ C_ash percolate_)/(C_ash solid −_ C_ash percolate_), (1)

where C_ash total_ is the ash content of the total digestate (calculated from the inputs and outputs during the experiment), C_ash percolate_ is the ash content of the percolate, and C_ash solid_ is the ash content of the solid digestate. After the total fresh mass fractions of the solid digestate and the percolate had been calculated, the amount of each mass component in the fresh matter of the materials was calculated. Lastly, the mass changes (e.g., VS loss and N mineralisation) and mass transfer between the solid biomass and the percolate were calculated.

A pilot-scale digestate storage experiment was conducted with the solid digestate obtained from the FB + HM Pilot I experiment. TS, VS, and nitrogen contents were analysed at the beginning and end of the storage experiment, and mass losses were calculated assuming that the mass of ash did not change during storage. Outdoor air temperature data during the storage experiment were obtained from a nearby (distance, 1.5 km) weather station [22].

### 2.4. Farm-Scale Experiments

#### 2.4.1. Monitoring Biogas Plant Operation

The biogas plant on the Knehtilä farm in Hyvinkää, South Finland consists of two mesophilic batch leach-bed reactors (total volume, 1000 m^3^; working volume, 810 m^3^ each) and a single (shared) percolation tank with a liquid volume of 200 m^3^. Biogas was collected from both the silos and the percolation tank. Temperature in the reactors was kept in the range of 33–36 °C. The biogas plant started operation in September 2018. The monitoring of the plant started during the third batch (Reactor 1: 11 March–8 July 2019, 117 days, referred to as Farm I) and continued with the fifth batch (Reactor 1: 12 July 2019–13 January 2020, 185 days, referred to as Farm II). Biogas production was only monitored for Reactor 1. The percolation tank was shared between the two reactors; thus, the contents of the percolation liquid were not reactor-/batch-specific. The amount of the percolation liquid circulating through each reactor silo was 100 m^3^. After digestion, the solid digestate was covered with a tarpaulin and stored in a stack on a concrete slab before field application. The Farm I digestate was stored for 26 days (8 July–3 August 2019) before application, while the Farm II digestate was stored for 125 days (13 January 2019–18 May 2020).

#### 2.4.2. Data Collection

Data were obtained from the biogas process with the online measurements (control system of the plant) of volumetric biogas production and methane content, and through sampling the raw materials, fresh digestates, digestates after storage in the stacks, and the percolation liquid (TS, VS, N_tot_, NH_4_–N, BMP, RMP). One sample was taken from each solid substrate (CGS, CM, HM). Three parallel samples were taken from the digestates; 1 parallel sample consisted of 10 subsamples.

#### 2.4.3. Calculation of Methane Production Potential and Biogas Production

Methane production potential for Farms I and II was calculated via multiplying the amount of the substrates (Table 3) by the measured BMPs (Table A1, Appendix A). There were, however, some uncertainties in these results. First, the BMP analysis of the raw materials was based on samples from Farm I. Those BMP results were also used for the BMP calculation of Farm II. Second, biogas production measurements were missing for the period of 13 March–19 June 2019 at the beginning of the Farm I experiment. The biogas production of Farm I was, thus, calculated as follows: for a starting period of 14 days, biogas production was estimated via multiplying the methane production per VS during the starting period of Farm II by the VS content of Farm I. After 14 days, the methane production rate was stable for the rest of the process. The aim when running the plant was also to keep the biogas production constant by controlling the process parameters. The average production rate from the measuring period of 19 June–8 July 2019 was calculated and multiplied by the total duration of the experiment (excluding the starting period. The automation data, i.e., gas composition measurements of Farm II were obtained during the whole batch from Reactor 1 and the percolation-liquid tank. However, there was also some uncertainty regarding Farm II since the percolation-liquid tank was shared between Reactors 1 and 2. Thus, half of the biogas production measured from the percolation tank was originating from Reactor 1.

#### 2.4.4. Farm-Scale Mass Balance Calculations

The mass of the digestate was not measured, but calculated as follows. First, the mass of the remaining total solids (TSs) in the digestate was calculated with Equation (2) on the basis of the TS mass in the raw materials (Table 3), and methane (CH_4_) and carbon dioxide (CO_2_) production:

m_digestate_ = m_TS raw materials −_ (m_CH4_ + m_CO2_) (2)

Methane production was measured, and the amount of CO_2_ was calculated on the basis of the methane content of the produced biogas (Farm I: 56%; Farm II: 55%). After the mass of the remaining total solids (TS) in the digestate had been established, the mass of the digestate was calculated by dividing the TS mass with the analysed TS content (Table A2, Appendix A):

m_digestate_ = m_TS digestate/_C_TS digestate_, (3)

Furthermore, to calculate the RMP, the VS mass was calculated via multiplying the mass of the digestate by the analysed VS content (Table A2, Appendix A), and through multiplying the VS mass by the analysed RMP per VS (Table A1, Appendix A).

m_VS digestate_ = m_digestate_ × C_VS digestate_, (4)

RMP (m^3^ CH_4_) = m_VS digestate_ × RMP (m^3^CH_4_/t_VS_), (5)

The mass of nitrogen (Ntot, NH_4_–N) in the solid substrate mixture was calculated on the basis of the mass of the substrates (Table 3) and the analysed nitrogen contents (Table A2, Appendix A). The amounts of nitrogen in the digestates were calculated in the same way. Organic nitrogen was calculated by subtracting NH_4_–N from the total nitrogen.

### 2.5. Analyses

Methane yields were converted into normal conditions (0 °C, 101.32 kPa) according to the ideal gas law. In the BMP and the RMP experiments, the methane production of the inoculum was subtracted from the results containing both substrate and inoculum to achieve the methane production of the feedstock.

TS and VS in the pilot-scale experiments were analysed according to SFS 3008 [23]. For the percolation liquids in Pilot Experiment I, water content was also analysed via azeotropic distillation (at SYNLAB Analytics and Services Finland Oy) to account for the vaporisation of organic acids during TS analysis. In Pilot Experiment II, the VFA contents in all raw materials were analysed (Table 4); due to the relatively high VFA content in FB, VS was recalculated according to Vahlberg et al. [24] (Equations (6) and (7)). The same method was used to analyse the VS of the percolation liquid used in Pilot Experiment II.

(6) $$TS\left(\%\right)={TS}_{m}\left(\%\right)+\left(\frac{{VFA}_{volatility}(\%)}{100}\times {VFA}_{m}\left(\frac{mg}{L}\right)\times 10^{-4}\right),$$

(7) $$VS\left(\%\;of\;TS\right)=\frac{\left({TS}_{m}\left(\%\right)\times {VS}_{m}\left(\%\right)\right)+\left(10^{-4}{\times VFA}_{volatility}\left(\%\right)\times {VFA}_{m}(\frac{mg}{L})\right)}{TS(\%)},$$

where TS and VS are the calculated results, TS_m_ and VS_m_ are the measured values, VFA_volatility_ is an estimated volatility of 98% according to Vahlberg et al. [24], and VFA_m_ is the total VFA content measured in mg/L. In the pilot experiments, volatile fatty acids were determined using external standardisation, and an HP 6890 gas chromatograph with automatic injector HP 7683, an FID detector, and GC ChemStation Rev. B.03.02 (column HP-FFAP 10 m × 0.53 × 1.0 µm, carrier gas helium) [25]. Ammonia NH4 was analysed according to McCullough [26], and total nitrogen was analysed with an accredited inhouse method, Luke-JOK2001 Kjeldahl nitrogen, on the basis of the AOAC 984.13-1994 (1996) method [27] using Cu as a digestion catalyst, and the Foss Kjeltec 2400 Analyser Unit (Foss Tecator AB, Höganäs, Sweden).

Samples from the farm-scale experiments were analysed at Eurofins Finland: TS and VS were analysed according to EN13040: 2008 [28] and EN15935: 2012-11 [29], respectively Ammonia NH_4_ was analysed with the Kjeldahl method, VFA was analysed with an NIR spectrometer (Q Interline MB 3600, Q-Interline A/S, Tølløse, Denmark) according to EF2018 [30], and total nitrogen was analysed according to the EN 13654 [31] and EN 13342 [32] standards.

In the pilot experiments, the methane and carbon dioxide contents of biogas were analysed (in addition to online measurement) using a Combimass GA-m gas analyser (Binder Engineering GmbH, Ulm, Germany).

## 3. Results and Discussion

### 3.1. Pilot-Scale Experiments

#### 3.1.1. Pilot-Scale Methane Production

Pilot-Scale Experiment I was performed with FB without cosubstrates. In the FB Pilot I reactor, the pH of the percolation liquid dropped below 7, and NaHCO_3_ and NaOH were used to raise the pH. Eventually, methane production started when the pH increased to 7.5. The decrease in pH at the start-up phase is a typical problem for the solid digestion process [15,33]. The decrease in pH occurs when the easily degradable fraction of organic matter is hydrolysed and converted to volatile fatty acids (VFA). Although VFAs are intermediate products in the process, too high contents of VFAs result in a decrease in pH and the inhibition of methanogenesis.

**Figure 2:**
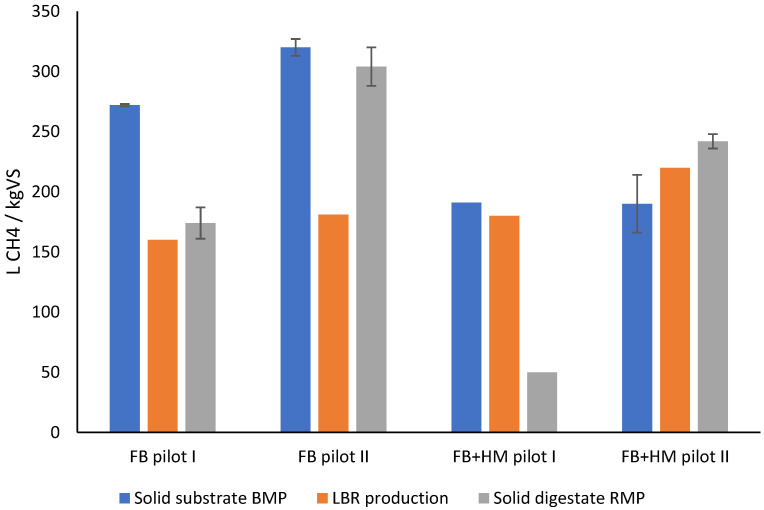
Specific methane production in pilot-scale reactors compared to the methane potential and the residual methane production of the solid substrates.

In FB Pilot I, the BMP of the whole crop fava bean was 272 L CH_4_/kg_VS_ (Table A1, Appendix A). When the specific methane production was calculated only through the inclusion of the VS of fava beans (VS of the percolation liquid excluded), methane production in the LBR reached 160 L CH4/kg_VS_ after 162 days. LBR methane production corresponded to 59% of the fava beans’ BMP (Figure 2), i.e., the LBR/BMP ratio was 59%. An alternative way of comparing LBR methane production to the BMP of raw materials is including the BMP (Table A1) and VS content (Table 2) of the percolate in the LBR-specific methane production and LBR/BMP ratio calculation. If calculated in this way, FB Pilot I would achieve a slightly lower LBR/BMP ratio of 55% (instead of 59%), since the average BMP of the FB and percolate weighted by VS content would be 258 L CH_4_/kg_VS_ (instead of 272), and the methane yield of the LBR would be 141 L CH_4_/kg_VS_ (instead of 160).

In FB Pilot II, the BMP of the fava beans was 320 L CH_4_/kg_VS_, while the LBR methane production reached 163 L CH_4_/kg_VS_ in 154 days, resulting in an LBR/BMP ratio of 57%. In the FB Pilot II experiment, the FB was dry and mouldy, which most likely affected methane production in the pilot reactor. If percolate VS and BMP were also included in the calculation, LBR methane production, weighted BMP, and the LBR/BMP ratio would be 169, 304, and 56%, respectively. In a laboratory study by Lehtomäki et al. [34] with a solid retention time of 55 days, the methane production of an LBR corresponded to 20% of the grass feedstock BMP; in a combined leach-bed–UASB process, the LBR/BMP ratio was 66%.

In the last pilot-scale experiment of the current study, the co-digestion of fava beans and horse manure was studied in the parallel reactors (FB + HM Pilots I and II). The BMPs of the total solid substrates (FB + HM) in the reactors were 191 and 190 L CH_4_/kg_VS_, respectively. At the beginning of the experiment, there was a gas leak in Reactor 1 that decreased the methane production. After the leak had been fixed, methane production was similar in the two reactors, reaching 180 in Reactor 1 and 220 L CH_4_/kg_VS_ in Reactor 2 in 133 days of digestion time. LBR/BMP ratios were 94 and 116%, respectively, meaning that the mix of fava beans and horse manure produced even more methane in Reactor 2 than that of the separate substrates in the BMP assays. If the VS and the BMP of the percolate were included in the calculation, the LBR/BMP ratios would be very similar (94 and 115%) because the percolate had very low VS content (Table 2). Riggio et al. [35] reached similar LBR/BMP ratios (85–99%) in the digestion of spent livestock bedding, but with the shorter digestion time of 60 days. The methane potentials of the whole crop fava-bean in the current study were lower than those previously reported in literature (387 L CH_4_/kg_VS_ for fava beans [36] and 440 L CH_4_/kg_VS_ for fava-bean straws [37]).

The amount of beans in relation to stem biomass could have varied between the pilot and BMP experiments, thus having an effect on biogas potential. However, the effect of the co-digestion of plant material with manure improved methane potential [33]. Even if the methane potential of manure were relatively low compared to the plant biomass, manure contains beneficial trace elements for anaerobic microorganisms and has the buffering capacity to maintain the process pH closer to neutral [38]. In addition, Degueurce et al. [39] showed that the microbial community in manure was strongly involved in methane production in an LBR process. The methane potential of horse manure in this study was relatively low (48 ± 9 L CH_4_/kg_VS_) and is highly dependent on bedding material [40], which was sawdust in this experiment.

The residual methane production (RMP) from the pilot-scale reactor digestates varied markedly in the different experiments and with different raw materials (Table A1, Appendix A). In the RMP experiments, inocula from the farm biogas reactor, mainly treating manure, were used. This might have affected the methane production of FB Pilot I and II digestates, as no manure or another cosubstrate was used in the pilot reactors. In the last experiment, the digestate RMP from the FB + HM Pilot I was markedly lower compared to previous digestates, but the digestate from FB + HM Pilot II was at the same level as that of previous ones. Although the digestates from the pilot reactors were mixed carefully, the differences in the RMP results from the last experiment may have been due to variation in the samples, as we could not measure exactly the same amount of FB and HM in a sample. The RMP from the percolation liquids decreased during the experiments (Table A1, Appendix A), which was expected, as the same liquid was used in all experiments with water dilution (percolation liquid having lower VS in each experiment, as shown in Table 1).

#### 3.1.2. Pilot-Scale Digestion Mass Balance

The mass balance was calculated for the FB + HM Pilot I experiment. After starting the experiment, 110 kg of water was added, and 58 kg of percolate and 35 kg of biogas were removed from the process. Hence, total mass increased by 4%, from 421 to 445 kg, during the process. According to the mass balance calculation, the mass of the solid material increased by 12% to 314 kg, while the mass of the percolate liquid decreased by 7% to 131 kg, indicating that part of the percolate was adsorbed to the solid material. A content comparison of the reactor and percolation tank at the beginning and end of the experiment showed that the VS mass and VS content of the solid raw material were reduced by 32 and 39%, while the VS mass and VS content of the percolate decreased by 30 and 25%, respectively (Table 4). The total VS mass (solid material and percolate combined) decreased by 32%, while the total VS content was reduced by 39%. The VS removal of the solid substrates was slightly lower than the VS removal reported for LBR processes fed with grass (34–55%, 55 days of digestion time) [34] and bedding-straw (45–60%, 110 days) [41] feedstocks. A comparison of the VS removal percentage with the literature values was complicated due to the addition of water and the removal of the percolate, which were performed in the current study.

According to the mass balance calculation, the masses of total N and NH_4_–N in the solid material were increased by 17 and 191%, respectively. The masses of the total N and the NH_4_–N in the percolate were increased by 61 and 39%, respectively (Figure 2). The mass of total N in the total material (solid material + percolate) was increased by 22% during digestion. The calculated increase in N could be attributed to inaccurate analyses and difficulties in sampling the heterogeneous solid biomasses.

**Figure 3:**
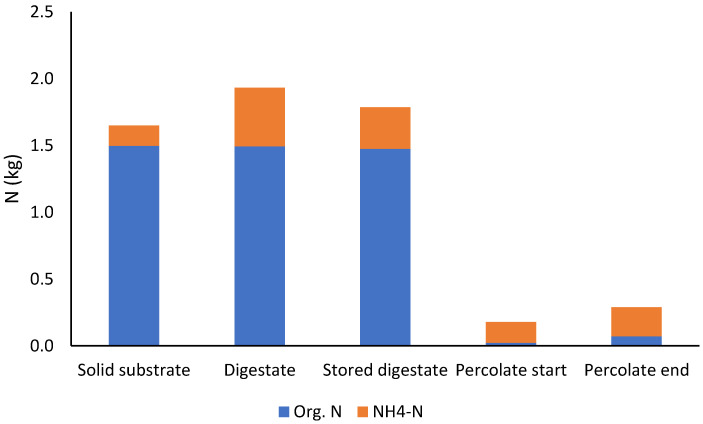
Masses of organic and NH_4_–nitrogen in solid substrate (fava bean + horse manure), percolation liquid, and digestate before and after storing in the FB + HM Pilot I experiment.

VS and nitrogen losses during simulated storage were tested in FB + HM Pilot I. The solid digestate was stored outside for 35 days (20 October–24 November 2020) in a 1 m^3^ metal reactor container without heating. The top of the container was only loosely covered with a tarpaulin, allowing for an exchange of gases between the porous digestate and air, probably resulting in (partial) aerobic composting conditions. During storage, the outside air temperature varied between −4.7 and 12.5 °C (average, 5.0 °C). The temperature of the digestate was not measured. The mass losses of VS, total N, organic N, and NH_4_–N during storage were 5, 8, 1, and 29%, respectively (Figure 3). Their contents were decreased by 8, 11, 4, and 31%, respectively, indicating that the (partially) aerobic digestate storage after a leach-bed process might cause nitrogen losses and greenhouse gas emissions. As the duration of batches in leach-bed reactors varies (see Section 2.4.1) and also affects the amount of organic material in the digestate, this experiment was only indicative, and more research is needed to ensure the sustainability of the process. Möller et al. [42] assumed a total N loss of 37.5% for the separated solids of the digestate during storage and handling, when it is turned several times before land application [42]. Further information about nitrogen losses during the storage of the solid digestate was not found in the literature. For comparison, over 20% of total N was lost in 33 days in a digestate solid aerobic composting experiment by Bustamante et al. [43].

### 3.2. Farm-Scale Experiments

**Figure 4:**
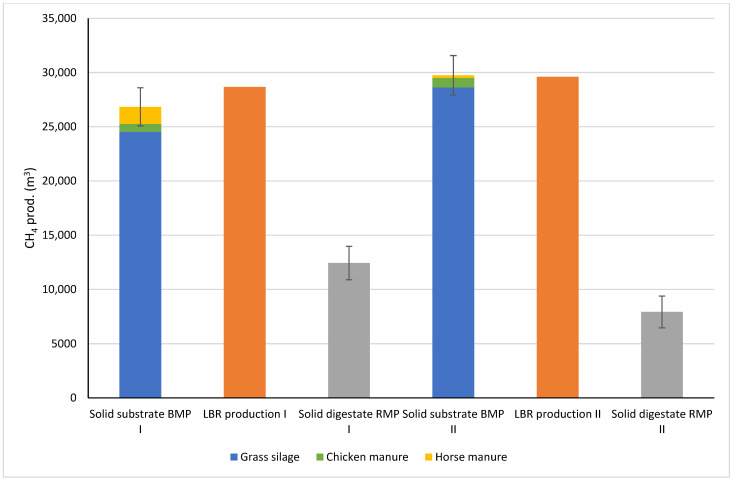
Methane production potentials of the solid substrates, LBR methane production, and residual methane production of the solid digestate in Farm I and II experiments.

On the basis of the raw material amounts (Table 3) and measured BMPs (Table A1, Appendix A), the total calculated methane production potentials of the solid substrates (excluding percolation liquid) were 26,834 m^3^ for Farm I and for 29,746 m^3^ Farm II (Figure 4). The use of local chicken (CM) and horse manure (HM) only slightly increased the production potential, but they might more significantly impact the realisation of methane production due to the beneficial properties of manure (suitable microorganisms, trace elements, buffering capacity) [38,39]. The realised methane production was 28,676 m^3^ (107% of the potential) and 29,607 m^3^ (100% of the potential). The LBR/BMP ratios were similar in the pilot co-digestion experiments. Chiumenti et al. [18] realised high methane production potential when cow manure and other agricultural biomass types (maize, ryegrass, alfalfa, and triticale silages, and straw) were used as substrates in a full-scale LBR plant. They further compared the process with full-scale wet digestion and concluded that the LBR process could achieve the same biogas production as that of wet digestion.

The residual BMP (RMP) measured from the digestate (Figure 4, Table A2, Appendix A) was 46% of the raw material BMP in Farm I and 27% in Farm II. The retention time in dry digestion clearly had an effect on the RMP. With the longer retention time of 185 days in Farm II, RMP was nearly halved compared to Farm I. However, the RMP in this experiment did not describe the unused BMP left in the batch because a new inoculum was added for RMP assessment, but it indicates the possibility of methane emissions during open storage.

**Figure 5:**
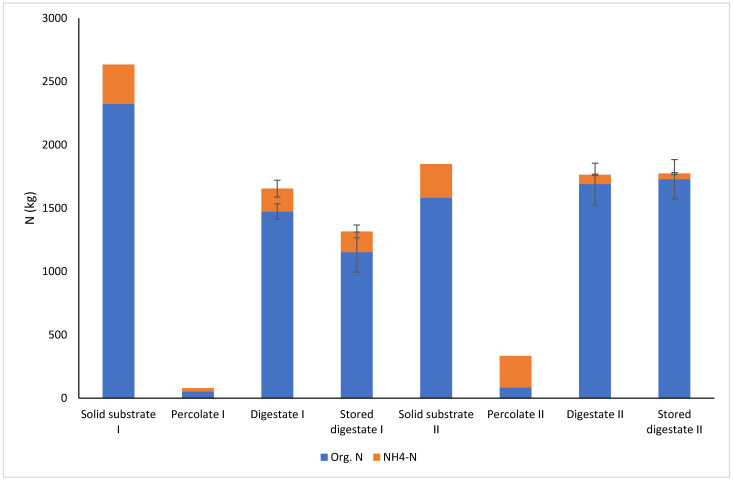
Organic N and NH_4_–N in the solid substrates, percolation liquid, and fresh and stored solid digestates in the Farm I and II experiments.

Even though the amount of total nitrogen should not markedly change during AD, only 63% of total nitrogen was left in the digestate in Farm I (Figure 5). This could be partly explained with the degradation of organic nitrogen into NH_4_–N and the transfer of NH_4_–N from the bed to the percolation liquid. However, a question arises regarding the low N content in the percolation liquid. One explanation comes from the sampling, which caused uncertainty to the results. Only one sample and no replicates were taken from the raw materials. Another explanation for the loss of N was the Farm I experiment ending in July on a warm summer day (max +18 °C). After the silo had been opened, it was emptied with a front loader into a storage stack, and the samples were taken from the stack and not directly from the silo. Thus, NH_4_–N in the digestate was most likely evaporated from the samples, as was seen in the simulated storage experiment on the pilot scale (Section 3.1.2). Furthermore, the total nitrogen content was decreased during storage in a stack. Lastly, only 50% of the total nitrogen in the raw materials was left in the digestate after three weeks of storage. According to the review by Möller and Müller [9], the separated solids of slurry digestate with a relatively high mineral N content have a high potential for N losses, particularly via ammonia volatilisation, leaching, and gaseous loses through denitrification after nitrification during partially aerobic storage and handling. Möller et al. [42] estimated an N loss of 37.5% for the separated solids of the digestate in German biogas plants.

In the Farm II experiment, nitrogen was better restored in the digestate. A total of 95% of the total nitrogen was left in the fresh digestate, and the N content was not decreased during storage. The conditions for the opening of the reactor silo in Farm II and during the storage of the digestate were totally different compared to those of Farm I. The Farm II experiment was completed in January, and the silo was emptied on a cold winter day (max +1 °C); storage was during winter and spring (January–May). Another reason for the good preservation of N during storage was the relatively low RMP in the digestate due to the long retention time in the Farm II experiment. For comparison, 8% of the total N was lost in the pilot-scale 35-day storage experiment at 5 °C (see Section 3.1.2). A high RMP, as in Farm I, indicated that there was still easily degradable organic matter left in the digestate, which enhanced composting in the stack after the conditions had been changed into aerobic. Furthermore, during composting, nitrogen is lost through ammonia volatilisation, nitrification, and denitrification processes. These potential nitrogen losses pose the threat of losing the benefits of biogas production that converts green manure into mobile fertilisers, thereby allowing for the better allocation of nutrients in the farming system [5,6].

## 4. Conclusions

Leach-bed reactor (LBR) technology is a promising option for biogas production from solid agricultural biomass on the pilot and farm scales. On the pilot scale, both the quality of the raw material and the operation procedures affected methane production. The co-digestion of fava beans with manure resulted in higher methane production than the sum of the individual biogas production potentials of the raw materials, indicating that manure improved the process efficiency. The summertime storage experiment indicated a potential risk for nitrogen losses. However, more research is needed to show the correlation between residual organic matter content and eventual nitrogen loss, and other parameters having an effect on nitrogen loss.

In both farm-scale experiments, methane production was equal to or higher than the methane production potentials of the raw materials, but variations in nitrogen losses during processing were high. For farmers, energy production is only part of the expectations regarding biogas plants, and the other part is high-quality fertilisers with high nitrogen content improving crop yields. On the basis of the results of this work, the three most important factors for preserving nitrogen content are cold weather during opening the silo at the end of the batch, the long retention time of the experiment, and low temperature during the storage of the digestate. In future studies, the reliability of the results could be improved by taking more samples for mass balance measurements. Digestion experiments could be improved by using replicate digesters.

To maintain a high nitrogen content in the digestate, careful planning and good management practices are important. In order to minimise greenhouse gas emissions and maximise fertiliser quality, the digester silo should not be opened during the warmest period of the summer, and the retention time should be long enough to ensure the full degradation of the substrates.

## Figures and Tables

**Table 1:** Substrate/inoculum VS:VS ratios used in BMP and RMP experiments for the raw materials of the pilot- and farm-scale experiments.

Raw Material	VS_substrate_:VS_inoculum_
FB pilot I and II	0.5
FB pilot III	0.75
HM pilot	0.75
HM	0.5
CM	0.5
Pilot percolation liquid	0.3 and 0.75
Pilot digestate RMP	0.5
Farm-scale digestate RMP	0.5 and 2.0
Pilot percolation liquid RMP	0.1 and 0.5

**Table 2:** Initial mass of the solid substrates and the percolation liquids in the pilot experiments.

Feed	FB Pilot I			FB Pilot II		
	kg_FM_^1^	kg_TS_^2^	kg_VS_^3^	kg_FM_	kg_TS_	kg_VS_
Fava beans	260	66	63	230	49	41
Percolate	150	11	9	150	5	3
	FB + HM pilot I			FB + HM pilot II		
Fava beans	230	65	58	230	65	58
Horse manure	51	16	15	52	16	15
Percolate	140	2	1	150	2	1

^1^ Mass of fresh matter in kilograms; ^2^ mass of TS; ^3^ mass of VS.

**Table 3:** Initial masses of the solid substrates in the farm experiments.

Feed	Farm I			Farm II		
	t_FM_^1^	t_TS_^2^	t_VS_^3^	t_FM_	t_TS_	t_VS_
CGS	360	104	96	353	119	111
CM	18	19	17	22.5	3	3
HM	56	7	5	7.5	15	6
Sum	434	130	118	383	137	120

^1^ Mass of fresh matter in tonnes; ^2^ TS mass; ^3^ VS mass.

**Table 4:** Contents of the materials at the beginning and end of the FB + HM Pilot I experiment.

	TS (%)	VS (%)	N_tot_ (kg/t)	NH_4_–N (kg/t)
Solid raw material (FB + HM)	28.91	25.94	5.87	0.54
Digestate	18.08	15.70	6.16	1.40
Digestate after storage (35 d)	16.71	14.41	5.51	0.96
Percolate start	1.82	0.69	1.28	1.12
Percolate end	1.90	0.52	2.20	1.66

## Data Availability

The data presented in this study are available on request from the corresponding author. Provision of the data for a third party needs to be agreed separately.

## Appendix A

**Table A1:** BMP and RMP results from pilot and farm experiments.

	BMP L CH_4_/kg_VS_	VS (%)
FB pilot I/FB	272 ± 1	48
FB pilot II/FB	320 ± 7	48
FB + HM pilot I and II/FB	227 ± 28	51
FB + HM pilot I and II/HM	48 ± 9	51
FB pilot I/percolation liquid	160 ± 11	48
FB pilot II/percolation liquid	74 ± 22	48
FB + HM pilot I and II/percolation liquid	57 ± 16	51
Farm I and II/CGS	256 ± 15	67
Farm I and II/HM	93 ± 14	67
Farm I and II/CM	150 ± 18	67
	RMP L CH_4_/kg_VS_	
FB pilot I/digestate	174 ± 13	48
FB pilot II/digestate	304 ± 16	88
FB + HM pilot I/digestate	50 ± 6	55
FB + HM pilot II/digestate	242 ± 56	55
FB pilot I/percolation liquid	373 ± 188	88
FB pilot II/percolation liquid	128 ± 82	88
FB + HM pilot I/percolation liquid	14 ± 4	55
FB + HM pilot II/percolation liquid	12 ± 6	55
Farm I/digestate	218 ± 27	67
Farm II/digestate	136 ± 25	51

**Table A2:** Analysis results from Farm I and II experiments.

Experiment	Sample	TS (%)	VS (%)	N_tot_ (kg/t)	NH_4_–N (kg/t)
Farm I	Digestate (117 days)	22.7 ± 0.5	19.8 ± 0.2	6.66 ± 0.01	0.63 ± 0.23
	Digestate after storage (26 days)	36.1 ± 1.3	32.1 ± 1.9	8.78 ± 0.04	0.90 ± 0.28
Farm II	Digestate (185 days)	20.1 ± 2.2	17.0 ± 2.0	6.58 ± 0.07	1.07 ± 0.08
	Digestate after storage (125 days)	17.4 ± 2.9	15.1 ± 2.9	5.90 ± 0.08	0.66 ± 0.11
Percolation liquid	Sample 1 (13 March 2019)	1.8		1.58	0.90
	Sample 2 (8 July 2019)	2.0	0.9	2.20	1.18
	Sample 3 (14 January 2020)	1.7		2.21	1.35

## References

[1] United Nations Framework Convention on Climate Change The Paris Agreement. https://unfccc.int/process-and-meetings/the-paris-agreement.

[2] European Commission. https://commission.europa.eu/strategy-and-policy/priorities-2019-2024/european-green-deal_en.

[3] European Council Fit for 55. https://www.consilium.europa.eu/en/policies/green-deal/fit-for-55-the-eu-plan-for-a-green-transition/.

[4] International Energy Agency World Energy Outlook 2022. https://iea.blob.core.windows.net/assets/830fe099-5530-48f2-a7c1-11f35d510983/WorldEnergyOutlook2022.pdf.

[5] Winquist E., Rikkonen P., Pyysiäinen J., Varho V. (2019). Is biogas an energy or a sustainability product?—Business opportunities in the Finnish biogas branch. J. Clean. Prod..

[6] Marttinen S., Luostarinen S., Winquist E., Timonen K. (2015). Rural Biogas: Feasibility and Role in Finnish Energy System, BEST Suitable Bioenergy Solutions for Tomorrow.

[7] Koppelmäki K., Parviainen T., Virkkunen E., Winquist E., Schulte R.P.O., Helenius J. (2019). Ecological intensification by integrating biogas production into nutrient cycling: Modeling the case of Agroecological Symbiosis. Agric. Syst..

[8] Möller K., Stinner W., Deuker A., Leithold G. (2008). Effects of different manuring systems with and without biogas digestion on nitrogen cycle and crop yield in mixed organic dairy farming systems. Nutr. Cycl. Agroecosyst..

[9] Möller K., Müller T. (2012). Effects of anaerobic digestion on digestate nutrient availability and crop growth: A review. Eng. Life Sci..

[10] Siegmeier T., Blumenstein B., Möller D. (2015). Farm biogas production in organic agriculture: System implications. Agric. Syst..

[11] Stinner W., Möller K., Leithold G. (2008). Effects of biogas digestion of clover/grass-leys, cover crops and crop residues on nitrogen cycle and crop yield in organic stockless farming systems. Eur. J. Agron..

[12] Koppelmäki K., Lamminen M., Helenius J., Schulte R.P.O. (2021). Smart integration of food and bioenergy production delivers on multiple ecosystem services. Food Energy Secur..

[13] Nordberg Å., Jarvis Å., Stenberg B., Mathisen B., Svensson B. (2007). Anaerobic digestion of alfalfa silage with recirculation of process liquid. Bioresour. Technol..

[14] Van D.P., Fujiwara F., Tho B.L., Toan P.P.S., Minh G.H. (2020). A review of anaerobic digestion systems for biodegradable waste: Configurations, operating parameters, and current trends. Environ. Eng. Res..

[15] Jha A.K., Li J., Nies L., Zhang L. (2011). Research advances in dry anaerobic digestion process of solid organic wastes. Afr. J. Biotechnol..

[16] Karthikeyan O.P., Visvanathan C. (2013). Bio-energy recovery from high-solid organic substrates by dry anaerobic bio-conversion processes: A review. Rev. Environ. Sci. Biotechnol..

[17] Bayrakdara A., Sürmelia R.Ö., Çallia B. (2018). Anaerobic digestion of chicken manure by a leach-bed process coupled with side-stream membrane ammonia separation. Bioresour. Technol..

[18] Chiumenti A., da Borso F., Limina S. (2018). Dry anaerobic digestion of cow manure and agricultural products in a full-scale plant: Efficiency and comparison with wet fermentation. Waste Manag..

[19] Riya S., Meng L., Wang Y., Lee C.G., Zhou S., Toyota K., Hosomi M., Abomohra A. (2020). Dry Anaerobic Digestion for Agricultural Waste Recycling. Biogas—Recent Advances and Integrated Approaches.

[20] Demirer G.N., Chen S. (2008). Anaerobic biogasification of undiluted dairy manure in leaching bed reactors. Waste Manag..

[21] Breitenbeck G.A., Schellinger D. (2004). Calculating the Reduction in Material Mass And Volume during Composting. Compost Sci. Util..

[22] Finnish Meteorological Institute Temperature Data for Jokioinen Ilmala. https://en.ilmatieteenlaitos.fi/download-observations.

[23] (1990). Veden, Lietteen ja Sedimentin Kuiva-Aineen ja Hehkutusjäännöksen Määritys: Standardi (In Finnish, A Standard for Determination of Dry Matter and Volatile Solids for Water, Slurry and Sediment).

[24] Vahlberg C., Nordell E., Wiberg L., Schnürer A. Method for Correction of VFA Loss in Determination of Dry Matter in Biomass. SGC Rapport, 2013, p. 273. http://www.sgc.se/ckfinder/userfiles/files/SGC273.pdf.

[25] Tampio E., Ervasti S., Paavola T., Heaven S., Banks C., Rintala J. (2014). Anaerobic digestion of autoclaved and untreated food waste. Waste Manag..

[26] McCullough H. (1967). The determination of ammonia in whole blood by a direct colorimetric method. Clin. Chim. Acta.

[27] (1996). Protein (Crude) in Animal Feed and Pet Food.

[28] (2008). Soil Improvers and Growing media—Sample Preparation for Chemical and Physical Tests, Determination of Dry Matter Content, Moisture Content and Laboratory Compacted Bulk Density.

[29] (2012). Sludge, Treated Biowaste, Soil and Waste—Determination of Loss on Ignition.

[30] (2018). In-House Method EF 2018, IC-Technique.

[31] (2001). Soil Improvers and Growing Media. Determination of Nitrogen.

[32] (2000). Characterization of Sludges—Determination of Kjeldahl Nitrogen.

[33] Maritza M.C., Zohrab S., Hanson A., Smith G., Funk P., Yu H., Longworth J. (2008). Anaerobic digestion of municipal solid waste and agricultural waste and the effect of co-digestion with dairy cow manure. Bioresour. Technol..

[34] Lehtomäki A., Huttunen T., Lehtinen T.M., Rintala J.A. (2008). Anaerobic digestion of grass silage in batch leach bed processes for methane production. Bioresour. Technol..

[35] Riggio S., Torrijos M., Debord R., Esposito G., van Hullebusch E.D., Steyer J.P., Escudié R. (2017). Mesophilic anaerobic digestion of several types of spent livestock bedding in a batch leach-bed reactor: Substrate characterization and process performance. Waste Manag..

[36] Pakarinen A., Maijala P., Jaakkola S., Stoddard F.L., Kymäläinen M., Viikari L. (2011). Evaluation of preservation methods for improving biogas production and enzymatic conversion yields of annual crops. Biotechnol. Biofuels.

[37] Petersson A., Thomsen M., Hauggaardnielsen H., Thomsen A. (2007). Potential bioethanol and biogas production using lignocellulosic biomass from winter rye, oilseed rape and faba bean. Biomass Bioenergy.

[38] Thamsiriroj T., Nizami A.S., Murphy J.D. (2012). Why does mono-digestion of grass silage fail in long term operation?. Appl. Energy.

[39] Degueurce A., Tomas N., Le Roux S., Martinez J., Peu P. (2016). Biotic and abiotic roles of leachate recirculation in batch mode solid-state anaerobic digestion of cattle manure. Bioresour. Technol..

[40] Hadin Å., Eriksson O. (2016). Horse manure as feedstock for anaerobic digestion. Waste Manag..

[41] Torres-Castillo R., Llabrés-Luengo J., Mata-Alvarez J. (1995). Temperature effect on anaerobic digestion of bedding straw in a one phase system at different inoculum concentration. Agric. Ecosyst. Environ..

[42] Möller K., Schultz R., Müller T. (2010). Substrate inputs, nutrient flows and nitrogen loss of two centralized biogas plants in southern Germany. Nutr. Cycl. Agroecosyst..

[43] Bustamante M.A., Alburquerque J.A., Restrepo A.P., de la Fuente C., Paredes C., Moral R., Bernal M.P. (2012). Co-composting of the solid fraction of anaerobic digestates, to obtain added-value materials for use in agriculture. Biomass Bioenergy.

